# Multiple myeloma and Chagas disease: qPCR as a marker for preemptive antiparasitic therapy: a case reports series and review

**DOI:** 10.1590/S1678-9946202466010

**Published:** 2024-02-05

**Authors:** Noemia Barbosa Carvalho, Vera Lúcia Teixeira de Freitas, Fernanda Salles Seguro, Rita Cristina Bezerra, Giancarlo Fatobene, Érika Yoshie Shimoda Nakanishi, Helena Visnadi, Gracia Martinez, Marjorie Vieira Batista, Vanderson Rocha, Frederico Luis Dulley, Sílvia Figueiredo Costa, Maria Aparecida Shikanai-Yasuda

**Affiliations:** 1 Universidade de São Paulo, Faculdade de Medicina, Hospital das Clínicas, Divisão de Moléstias Infecciosas e Parasitarias, São Paulo, São Paulo, Brazil; 2 Universidade de São Paulo, Faculdade de Medicina, Departamento de Moléstias Infecciosas e Parasitarias, São Paulo, São Paulo, Brazil; 3 Universidade de São Paulo, Faculdade de Medicina, Hospital das Clínicas, Laboratório de Investigação Médica em Imunologia (LIM-48), São Paulo, São Paulo, Brazil; 4 Universidade de São Paulo, Faculdade de Medicina, Hospital das Clínicas, Serviço de Hematologia, Transfusão e Terapia Celular, São Paulo, São Paulo, São Paulo, Brazil; 5 Universidade de São Paulo, Faculdade de Medicina, Hospital das Clínicas, Laboratorio de Investigação Médica em Patogenese e Terapia Celular Dirigida em Onco-Imuno-Hematologia (LIM-31), São Paulo, São Paulo, Brazil; 6 Universidade de São Paulo, Faculdade de Medicina, Hospital das Clínicas, Laboratório de Investigação Médica em Parasitologia (LIM-46), São Paulo, São Paulo, Brazil; 7 AC Camargo Cancer Center, Departamento de Infectologia, São Paulo, São Paulo, Brazil; 8 Universidade de São Paulo, Faculdade de Medicina, Hospital das Clínicas, Laboratório de Investigação Médica em Protozoologia (LIM-49), São Paulo, São Paulo, Brazil; 9 Universidade de São Paulo, Faculdade de Medicina, Instituto de Medicina Tropical de São Paulo, São Paulo, São Paulo, Brazil

**Keywords:** Multiple myeloma, Chagas disease, *T. cruzi* parasitemia, Conventional PCR, Quantitative PCR

## Abstract

Multiple myeloma (MM) associated with Chagas disease is rarely described. This disease and its therapy suppress T cell and macrophage functions and increase regulatory T cell function, allowing the increase of parasitemia and the risk of Chagas Disease Reactivation (CDR). We aimed to analyze the role of conventional (cPCR) and quantitative Polymerase Chain Reaction (qPCR) for prospective monitoring of *T. cruzi* parasitemia, searching for markers of preemptive antiparasitic therapy in MM patients with Chagas disease. Moreover, we investigated the incidence and management of hematological diseases and CDR both inside and outside the transplant setting in the MEDLINE database. We found 293 studies and included 31 of them. Around 1.9–2.0% of patients with Chagas disease were reported in patients undergoing Stem Cell Transplantation. One case of CDR was described in eight cases of MM and Chagas disease**.** We monitored nine MM and Chagas disease patients, seven under Autologous Stem Cell Transplantation (ASCT), during 44.56±32.10 months (mean±SD) using parasitological methods, cPCR, and qPCR. From these patients, three had parasitemia. In the first, up to 256 par Eq/mL were detected, starting from 28 months after ASCT. The second patient dropped out and died soon after the detection of 161.0 par Eq/mL. The third patient had a positive blood culture. Benznidazole induced fast negativity in two cases; followed by notably lower levels in one of them. Increased *T. cruzi* parasitemia was related to the severity of the underlying disease. We recommend parasitemia monitoring by qPCR for early introduction of preemptive antiparasitic therapy to avoid CDR.

## INTRODUCTION

Multiple myeloma is a B-cell malignancy associated with uncontrolled plasma cell proliferation that evades bone marrow immune surveillance, leading to anemia, lytic lesions, and increased secretion of monoclonal immunoglobulin, which is associated with kidney dysfunction and hypercalcemia^1^.

Plasma cell dysfunction shown in the interactions with dendritic, bone marrow stromal cells, natural killer, and T cells is mediated by myeloid-derived suppressor cells and cytokines, such as IL-10, TGFβ, and IL-6, down-regulating antigen recognition and processing and up-regulating T reg cells^2^. Even before symptoms emerge, clonal plasma cells interact with the bone marrow microenvironment, inducing an immunosuppressive state and increasing the risk for infections^3-5^.

The preferred first-line regimen for newly diagnosed patients with multiple myeloma (MM) includes induction therapy (triple or quadruple combination), autologous stem cell transplantation (ASCT) for eligible individuals, and maintenance therapy until progression^6^. The therapy involves a combination of agents^7-12^, including steroids, alkylating agents (melphalan and cyclophosphamide)^7,8^, immunomodulatory drugs (thalidomide and lenalidomide)^9^, proteasome inhibitors (PI, bortezomib)^10,11^, and anti-CD38 monoclonal antibodies (anti-CD38, anti-BCMA [B-cell maturation antigen] or anti-SLAMF-7 [Signaling lymphocytic activation molecule F7]^11-13^, bispecific T-cell engagers^14^, and chimeric antigen receptor T-cell therapy)^15^. These therapies aim to engage the patient’s immune response against clonal plasma cells. Although the medication used may temporarily impair the immune response, the long-term recovery of immune functions plays a crucial role in achieving good responses. However, B-cell suppression (hypogammaglobulinemia) is prolonged and may require immunoglobulin (Ig) replacement therapy^16^.

Around 1.9–2.0% of patients undergoing ASCT and Allogeneic Hematopoietic Stem Cell Transplantation (HSCT), reported in two series of 1,328 and 234 patients, had Chagas disease^17,18^. Furthermore, MM has been rarely reported in association with Chagas disease^18-22^.

Chagas disease is a neglected tropical disease caused by *Trypanosoma cruzi* and affects approximately 6 million people, mainly in Latin America^23^. Globalization, urbanization, and immigration from endemic areas to all the continents spread the disease worldwide^23^. The main transmission mechanisms are vectorial transmission, blood transfusion, organ transplantation, food contamination, congenital transmission, and laboratory accidents^24^. The acute phase is asymptomatic in most infected people and is followed by a chronic phase, which are classified as indeterminate form without cardiac or digestive involvement (70%), cardiac (20%), digestive (megaesophagus, megacolon, or both) (10%), and associated cardiac and digestive form (5%)^24^.

The balanced host-parasite interaction in chronic Chagas disease can be disrupted due to immune dysfunctions observed in MM^25,26^ and by immunosuppressive therapy or transplantation, representing an additional risk for Chagas disease reactivation (CDR)^25,26^. CDR is characterized by severe myocarditis and/or meningoencephalitis and, less frequently, by mild disease^25,27^.

In this study, we reported nine new cases of MM associated with Chagas disease, seven of whom undergoing ASCT, aiming to analyze the role of parasitemia monitoring by quantitative molecular method as a marker for preemptive antiparasitic treatment to prevent CDR. In addition, considering the severity and high fatality of CDR in hematological immunocompromised patients, we reviewed the occurrence of Multiple myeloma concomitantly with Chagas disease, as well as CDR under transplantation and hematological malignancies. We aimed to update the knowledge on diagnosis, treatment, management, and outcome of CDR in these patients.

## MATERIALS AND METHODS

### Patients

This study included nine MM patients with chronic Chagas disease, followed up from 2004 to 2022 at the Service of Hematology, Transfusion, and Cell Therapy, and the Infectious and Parasitic Diseases Clinic of Hospital das Clinicas da Faculdade de Medicina, University of Sao Paulo. From these, seven received autologous stem cell transplantation.

### Chagas disease and Chagas disease reactivation diagnoses

*T. cruzi* infection was diagnosed by two different positive serologies (Enzyme immunoassay [ELISA], indirect immunofluorescence, and/or chemiluminescence)^24^. For parasitemia monitoring, we employed molecular^28^and indirect parasitological enrichment methods (blood culture, xenodiagnoses, or both), as well as weekly direct microscopic techniques in blood^25,27^ during the immediate pre-transplantation period. In ASCT, this protocol was followed weekly for at least two months after transplantation. In Allogeneic SCT, weekly analyses are conducted during the first two months post-transplant; monthly analysis for up to six months; every trimester for one year. Moreover, in Allogeneic SCT, analyses are performed every six months throughout the immunosuppression period. It returns to weekly protocol upon suspicion of CDR or increased immunosuppression or Graft-versus-Host-disease. For the diagnosis of CDR, parasites in the peripheral blood were diagnosed by direct microscopy via Microhematocrit (MicroHt), Buffy coat, or histopathology of possible inflammatory lesions containing amastigotes^25,27^.

### Conventional PCR and quantitative PCR

cPCR was performed using the S35/36 kinetoplast primer pair, which amplifies the *T. cruzi* minicircle 330-base pair (bp) sequence^28^. As controls for the presence of DNA amplification inhibitors, duplicate patient’s samples containing parasite DNA were used. For qPCR, the TCZ3/TCZ4 nuclear primer pair, which amplifies the 149 bp microsatellite sequence, was used^28^.

### Multiple myeloma management

Until 2021, the triple combination of cyclophosphamide, thalidomide, and dexamethasone (CTD) was the standard therapy for patients with newly diagnosed MM^29^. After the approval of bortezomib in the public setting, the combination of bortezomib, thalidomide, and dexamethasone (VTD) has been the preferred regimen^30^. After up to nine cycles of induction, consolidation with ASCT was performed with melphalan (140–200 mg/m^2^). For patients not achieving a very good partial response after transplant, thalidomide was prescribed as a maintenance therapy. One of the patients received IFN-γ as post-transplant maintenance^31^. However, this therapy did not improve overall survival and was permanently discontinued. Patients were classified following the International Staging System (ISS) and Durie-Salmon (DS) classifications^6,32^.

### Ethics statement

This study was approved by the Research Ethics Committee of the involved institution, Brazil (protocol numbers 095/1995 and 1043/07). All included patients, whether prospective or retrospective, signed an informed consent form. The inclusion of retrospectively collected data was approved, ensuring patients anonymity. Some retrospectively included patients did not sign it since they died or dropped out. All data were collected anonymously from medical records.

### Review on Chagas disease and multiple myeloma

In this review, two authors searched papers on the MEDLINE database published from 1981 to July 2023, using the terms: “Chagas disease and hematological malignancies,” “Chagas disease and Multiple myeloma,” “Chagas disease and leukemia,” “Chagas disease and lymphoma,” “Reactivation of Chagas disease,” Reactivation of Chagas disease and Multiple Myeloma,” “Reactivation of Chagas disease and lymphoma,” and “Reactivation of Chagas disease and leukemia.” From these primary searches, secondary references were added from 1968 to July 2023. A total of 276 articles and 17 secondary references were found, totaling 293 English, Spanish, and Portuguese publications. In total, 31 original articles were included, 25 regarding Myeloma and Chagas disease and Chagas disease reactivation, both in hematological malignancies and hematopoietic stem cell transplantation, and six on parasitemia detection in immunosuppressed patients with Chagas disease.

## RESULTS

### Case reports

A total of nine cases were monitored for *T. cruzi* parasitemia from 3 to 88 months (44.56±32.10; mean±SD); seven under ASCT and three without transplantation. All were Brazilian citizens, except Case 3, a Bolivian citizen.

### Case 1

A 51-year-old male patient was diagnosed with MM IgG in August 2004 with bone lytic lesions and renal dysfunction (ISS and laboratory data) (Table 1). He had essential hypertension and positive serology for *T. cruzi* antigens, referring to antiparasitic treatment with benznidazole 10 years before. He had chagasic myocardiopathy (Table 1).

**Table 1:** - *Trypanosoma cruzi* parasitemia monitoring by molecular and parasitological methods and clinical evolution in seven Multiple Myeloma + Chagas disease patients under Autologous Stem Cell Transplantation.

Case	Age at diagnosis Sex	MM diagnosis date, subtype, SPEP, ISS stage, DS	Lines of treatment (month/day/year)	Best response (IMWG criteria)	Status/date/cause	Chagas disease evaluation (clinical form)	cPCR/Hemoculture-H, Xeno-diagnosis-X	qPCR/ MicroHt or Buffy coat	Months of Parasitemia monitoring/total* (BNZ)
1	51 years Male	08/2004 IgG κ, SPEP: 1.2g/dL, ISS: stage2 DS IIIB	1^st^: Induction: high dose Dexa 3 cycles **ASCT: melphalan 200 mg/m2 -01/2005** Maintenance: interferon γ+ Dexamethasone until 07/2007	CR	01/2005 Pneumonia/06/2005 H. Zoster, Death 8/2019 - aortic aneurysm	Echocardiogram -hypokinesia VE, Left VE hypertrophy, ECG -Anterosuperior Division Block (Cardiac)	06/01/2004 N H0X0 03/29/2005 N H0X0 07/03/2007 N H0X0 02/26/2008 N H0X0 12/10/2008 N H0X0 05/01/2010 N H0X0 12/07/2010 N H0X0	U/N U/N U/N U/N U/N U/N U/N	78 months*
2	55 years Female	09/2004 Subtype, SPEP, ISS: unknown DS IIIB	1^st^ Induction: Melphalan + dexamethasone: 09/2004 - 04/2005 2^nd^ Induction: VAD 4 cycles: 04/05 – 09/05 **ASCT: Busulfan 12 mg/kg + melphalan 100 mg/m2 – 05/2006** Thalidomide: 07/2006 – 07/2010 Radiotherapy: 01/2010 – 04/2010 due to myeloma recurrence, 09/2010: Melphalan +Prednisone + Thalidomide Radiotherapy re-started in 12/2010	CR	Lost to follow-up on 04/2011	ECG changes in ventricular repolarization, Chest XR – normal, Holter 09/2008 isolated Supra-ventricular isolated arrhythmia, Echocardiogram – Diastolic dysfunction** (Significant non-specific ECG changes + Diastolic dysfunction)**	02/21/2006 PH0X0 07/25/2006 PH+X+ 10/09/2007P/H+X0 09/30/2008 PH+X+ 10/17/2008 PH+X+ 01/06/2009PH+X+ 03/03/2009 PH+X+ 03/12/2009 PH+X0 03/25/2009 NH+X+ 04/23/2009 NH0X0 08/25/2009 PH+X0 11/17/2009 NH+X0 04/23/2010 P 07/06/2010 P 01/18/2011 PH+X+	3.2/N 5.4//N 6.6/N 56.4/N 127.0/N 124.8/N 256.0/N 120.8/N U/N U/N 0.36/N U//N 0.07NA 10.9/NA 0.95/N	59 months* 28 months 28.5 months 31 months 33.5 months 03/07/2009 7 mg/kg/d –BNZ Exanthema 18^th^ day
3	55 years Female	07/2004 IgG λ, SPEP: 0.73 g/dL, ISS: stage 3 DS IIIA	1^st^:Induction: high dose Dexa 1 cycle + VAD 3 cycles 11/2004 – 02/2005 Maintenance: thalidomide until 01/2006 **ASCT: Busulfan 12 mg/kg + melphalan 100 mg/m2- 01/2006**	CR	Lost to follow-up on 06/ 2006	No cardiac abnormalities (Noncardiac)	08/30/2005 NH0X0 01/12/2006 NH0X0 02/01/2006 NH0X0 03/15/2006 N 04/07/2006 N	NA/N U/N U/N U/N U/NA	7 months*
4	37 years Female	Date: 11/2010 IgG κ, SPEP:2.5g/dL ISS: stage 2, DS IIIA	1st: CTD 9 cycles 12/2010 -08/2011 2^nd:^ Induction: CTD 4 × cycles 05/2015- 10/2015 **ASCT: melphalan 200mg/m2 – 04/2016** 3^rd^: CTD 6 cycles 01/2019 – 06/2019 4^th^: CD 8 cycles 01/2020- 08 2020 5^th^: CVAD 5 cycles 09//2020- 02/2021 6^th^: VD 7 cycles 05/2021-11/2021 7^th^: MD 4 cycles 05/2022 -08//2022	PR VGPR Stable Stable Stable PR Progression	Alive March 2023	Normal ECG and Chest XR, Contrast-enhanced esophageal XR - grade 1 dysmotility (Digestive form)	07/22/2014 NH0 01/05/2015 NH0 09/28/2015 NH0 04/25/2016 NH0 01/24/2017 N 06/22/2018 N 03/28/2019 N 10/19/2020 N 11/09/2021 N 03/08/2022N	U/N U/N U/N U/N NA/N NA/N NA/N NA/N NA/N NA/N	88 months* BNZ 08/2006 for 44 days due to Gastric intolerance and peripheral neuropathy
5	55 y Male	07/2013: IgG κ, SPEP: 2.04 /dL, ISS: stage 1, DS IIIA	1st: Induction: CTD 8 cycles 06/2014- 01/2015 2^nd :^Induction: CTD 10 cycles 02/2016 –11/2016 **ASCT: melphalan 200 mg/m2 -3/2017**	VGPR	Lost to follow-up on 06/2017	ECG- normal, Echo-cardiogram – Diastolic dysfunction, left auricular enlargement (Diastolic dysfunction)**	12/07/2015 N H0 03/28/2016 N H0 07/04/2016 N 12/12/2016 N 04/07/2017 N	U/N U/NA U/N NA/NA NA/N	52 months*
6	58 years Male	08/2015, IgG κ, SPEP: 3.6g/dL, ISS: stage 3, DS IIIA	1^st^ Induction: CTD 9 cycles 09/2015-05/2016 **ASCT: melphalan 200 g/m** ^**2**^ **08/2016** 2^nd^ Induction: CD 5 cycles01/2009-05/2009 5 cycles 01/2019-05/2019 **ASCT: melphalan 200g/m** ^**2**^ **07/2019** 3^rd^:CD 6 cycles 08/2020 -06/2021 4^th^ VCD 8 cycles 09/2021-03/2022	PR VGPR PR CR	Alive February 2023	ECG – normal 2016 and possible LV overload – 2019, 2015/2016 –Normal Echocardiogram, XR - EED (Significant non-specific ECG changes)	04/19/2016 N 04/25/2016 N 08/19/2019 N 09/04/2019 N	U/N U/N NA/NA NANA	42 months*
7	59 years Male	11/2015 IgG κ, SPEP: 2.5g/dL, ISS: stage 2, DS IIIA	1^st^: CTD 8 cycles 11/18/2015-07/17/2016 **ASCT: 01/12/2017** 2^nd^: CTD 9 cycles 03/25/2020-12/09/2020 3^rd^: VTD 8 cycles 11/09/2021 – 06/30/2022 ASCT: 01/12/2017	VGPR VGPR VGPR	Alive February 2023	ECG right branch block, Echo: diffuse hypokinesia, Diastolic dysfunction, discrete systolic dysfunction LVE EF 52% (Cardiac)	06/06/2016 N 07/04/2016 N 01/27/2017 N 08/08/2017 N 02/07/2020 N 11/16/2021 N	U/NA U//N NA/N NA/N NA/N NA/N	65 months*

MM = multiple myeloma; IMWG = International Myeloma Working Group; SPEP = serum protein electrophoresis; ISS = International Stating System; DS = Durie-Salmon Classification; MicroHt = microhematocrit; BNZ = benznidazole; ASCT =– autologous stem cell transplantation; ECG = electrocardiogram; CTD = cyclophosphamide, thalidomide, and dexamethasone; CR = complete response; PR = partial response; VGPR = very good partial response; VTD = bortezomib, thalidomide, and Dexamethasone; Dexa = dexamethasone; CAVD = cyclophosphamide, vincristine, doxorubicin, and dexamethasone; VD = bortezomib and dexamethasone; VCD = bortezomib, cyclophosphamide, and dexamethasone; MD = melphalan and dexamethasone; MDT = melphalan, dexamethasone, and thalidomide; VAD = vincristine, doxorubicin, dexamethasone; CD = cyclophosphamide, dexamethasone; EF = ejection fraction; LVE = left ventricular; H0 = negative blood culture; X0 = negative Xenodiagnosis; NA = not available; U = undetectable; XR EED = XR of the esophagus, stomach, and duodenum; *total period of parasitemia monitoring; **Diastolic dysfunction has been suggested as an early stage of chagasic myocardiopathy.

The patient received three cycles of induction with dexamethasone (480 mg per cycle), consolidation with high-dose melphalan (200 mg/m^2^), and underwent ASCT in January 2005. After the transplant, he achieved a complete response and received interferon and dexamethasone for maintenance until July 2007. The patient did not have any relapse until his death in August 2019 due to ruptured aortic aneurysm complications. During the follow-up, cPCR and direct microscopy in the blood, indirect parasitological blood cultures, and xenodiagnoses were negative (Table 1).

### Case 2

A 55-year-old female complained of back pain in August 2003. Work-up led to the diagnosis of MM with multiple lytic bone lesions. She underwent treatment with melphalan and dexamethasone, followed by four cycles of vincristine, doxorubicin, and dexamethasone (VAD), achieving a complete cure. Her pre-transplant evaluation evidenced seropositivity for *T. cruzi* antigens. She underwent ASCT on May 2006 with busulfan and melphalan. After the transplant, thalidomide was introduced and continued until September 2010. She presented significant nonspecific electrocardiographic changes and diastolic dysfunction on the echocardiogram, which has been suggested as an early expression of heart Chagas disease (Table 1). qPCR indicated low parasitemia before and after the transplant but it increased (56 par Eq/mL) 28 months post-transplant, representing 10× the mean of three pre-transplant samples (5.1 par Eq/mL). The highest number (256 par Eq/mL) was detected 33.5 months post-transplant, showing 50× the mean of par Eq/mL pre-transplant and 80× the lowest pre-transplant number of parasites Eq/ml. CDR was not confirmed by direct microscopy in the peripheral blood. Preemptive therapy with benznidazole at 7 mg/kg/d was introduced for 60 days. As an adverse event of benznidazole, she presented a maculopapular rash on her hands at 18 days of medication. Parasitemia decreased rapidly and remained at a low level during the months of follow-up. She was given radiotherapy in the lumbosacral spine in January, April, and December 2010. In September 2010, melphalan and prednisone were prescribed for a myeloma recurrence. Chagas parasitemia was monitored periodically (Table 1).

### Case 3

A 55-year-old female was diagnosed with IgG λ Lambda MM in July 2004 with lytic bone lesions (Table 1). She received one cycle of high-dose dexamethasone followed by five cycles of VAD and thalidomide maintenance, achieving complete remission. She underwent ASCT. Pre-transplant work-up showed a positive serology for *T. cruzi* antigens. No cardiac abnormalities were present. Parasitemia was monitored with PCR, but no positive parasitemia or reactivation was detected. She had an unremarkable clinical course and was referred to her primary hematologist, and she was lost to follow-up.

### Case 4

A 37-year-old female patient was diagnosed with MM, IgG κ in November 2010 after a non-traumatic vertebral fracture (Table 1). The only finding related to MM was multiple lytic bone lesions in the spine. Positive serology for *T. cruzi* antigens was observed at diagnosis. The patient had mild dysphagia and a digestive form of Chagas disease (Table 1). For the first line of therapy, the patient received six cycles of cyclophosphamide, thalidomide, and dexamethasone (CTD) with a complete response. After three years, she had a humerus fracture, and she received five cycles of CTD and consolidation with high-dose melphalan (200 mg/m^2^), achieving a very good partial response. In September and November 2014, parasitemia monitoring showed negative results (Table 1). She was prescribed benznidazole at 300 mg/day for 60 days before autologous stem cell transplantation in December 2015. On the 44^th^ day, benznidazole was interrupted due to peripheral neuropathy. Febrile neutropenia, mucositis grade IV, and diarrhea were noticed during the immediate post-transplant period. She successfully had neutrophil engraftment, and all adverse events were controlled. From June 2019 to October 2022, the patient had five relapses and was treated with different regimens, evolving as a stable disease. During all the follow-ups until 66 months post-transplant, cPCR maintained negative results (Table 1).

### Case 5

A 55-year-old male developed bone pain and was diagnosed with IgG κ MM affecting multiple sites of his lumbar vertebrae in July 2013. Due to impending spinal compression, he underwent urgent spinal decompression surgery with no neurologic sequelae (Table 1). He has been treated with eight cycles of CTD, starting from June 2014, plus radiotherapy, achieving a partial response. Pre-transplant work-up showed a positive serology for *T. cruzi* antigens, normal ECG, and diastolic dysfunction on echocardiogram, which has been suggested as an early change on chagasic cardiopathy. Due to disease progression, in February 2016, he restarted on CTD with 10 cycles, achieving a very good partial response. Parasitemia monitoring from December 2015 to April 2017 showed negative cPCR, qPCR, direct microscopy, and indirect parasitological exams (Table 1). He underwent ASCT in March 2017, and his post-transplant period was unremarkable. He was lost to follow-up in June 2017.

### Case 6

A 58-year-old man searched the healthcare service with a complaint of back pain for six months. He received the diagnosis of MM IgG κ (Table 1). He had a positive serology for *T. cruzi* antigens without cardiac or gastrointestinal symptoms. The first-line induction CTD regimen was started in September 2015, and the patient received nine cycles. The patient used dexamethasone as maintenance (20 mg per week) from June 2016 to August 2016. He received an ASCT in August 2016, achieving a partial response, and relapsed 26 months later. In January 2019, he started his second line of treatment with CD (cyclophosphamide 500 mg and dexamethasone 20 mg, weekly). Then, he received monthly cycles again until June 2019 and underwent a second ASCT until August 2019. He achieved a very good partial response after this regimen. In August 2020 and September 2021, new progression was detected, and the patient received new lines of treatment (VCD with bortezomib at 1.3 mg/m^2^, cyclophosphamide at 500 mg, and dexamethasone at 20 mg, weekly), as seen in Table 1, until April 2022. He remained on complete cure, with no evidence of clinically significant disease.

No symptoms of Chagas disease or positive parasitemia were reported during the hematological treatment.

### Case 7

A 59-year-old male was diagnosed with MM IgG κ with multiple bone lytic lesions in November 2015. The patient had positive serology for *T. cruzi* antigens and the Cardiac form of Chagas disease (Table 1). He received eight cycles of induction with CTD, achieving very good partial response and consolidation with high-dose melphalan (200 mg/m^2^) and ASCT in January 2017. Biological progression was detected two years after the ASCT. Second-line therapy was performed with the same regimen, with nine cycles of CTD until November 2020, achieving a very good partial response. A second progression was detected in October 2021, and a third-line regimen was initiated with VTD, followed by a very good partial response. Until October 2022, he was receiving thalidomide as maintenance with a sustained response. *T. cruzi* parasitemia monitoring since June 2016 showed negative results up to October 2022 (Table 1).

### Case 8

In 2013, a 57-year-old male was admitted to a local hospital due to lumbar and thoracic spine pain. He was found to have IgG κ MM with anemia, and multiple bone lesions (skull and vertebrae) (Table 2). He was treated with eight cycles of thalidomide and dexamethasone plus radiotherapy and referred to ASCT. At the pre-transplant evaluation in April 2015, he was found to have progressive disease, positive serology for hepatitis B and *T. cruzi* antigens*,* and was classified with the Indeterminate Form of Chagas disease (Table 2). In July 2015, he was started on VCD × two cycles and then four cycles of cyclophosphamide and dexamethasone (CD), with no response. His blood culture was positive but his sample’s cPCR and qPCR was unavailable. Starting in August 2015, he received benznidazole for 57 days. cPCR for *T. cruzi* was negative in August 2015, November 2015, and January 2016 (Table 2). Due to a lack of response for MM chemotherapy, the patient was referred back to his local practitioner and lost to follow-up.

**Table 2:** - *Trypanosoma cruzi* parasitemia monitoring by molecular/parasitological methods and clinical evolution of two patients with multiple myeloma and Chagas disease without transplantation.

Case	Age at diagnosis Sex	MM diagnosis date, subtype, SPEP, ISS stage, DS	Lines of treatment (month/day/ year)	Best response (IMWG criteria)	Status/date/ cause	Chagas disease evaluation (clinical form)	cPCR/ Hemoculture - H	qPCR/ DM	Parasitemia monitoring months/total* /(BNZ)
8	57 years Male	2014, IgG κ,SPEP 1,7 g/d, ISS: stage 1 DS III A	1^st^:TD 8 cycles 04/14 – 04/2015 Radiotherapy 04/24 – 05/2014 2^nd^: VCD - 2 cycles 07/15 – 08/15 CD - 4 cycles 09/15 – 12/15	Progressive	Lost to follow-up on 04/29/2016	Normal ECG, Echocardiogram, Chest/ EED XR (Indeterminate form)	08/18/2015 N/HP 08/28/2015 N/HNA 11/08/2015 N/HNA 01/26/2016 N/HNA	NA/N U/N U/N U/N	9 months* BNZ August 2015 57 days
9	60 years Male	08/2017, IgG λ,SPEP: 8.2 g/dL, ISS stage 3, DS III A	1^st^: CTD 8 cycles (12/2017-11/2018)	PR	Dead January/2019: Disease progression	Normal Echocardiogram (Digestive form)	09/12/2018 N/HNA 11/13/2018 P/HNA 12/14/2018 P/HNA	NA/NA 0.02/N 161/N	3 months* Lost to follow-up 12//2018

MM = multiple myeloma; SPEP = serum protein electrophoresis; ISS = International Staging System; DS = Durie-Salmon classification; IMWG = International Myeloma Working Group; DM = direct microscopy; CTD = cyclophosphamide, thalidomide, and dexamethasone; PR = partial response; TD = thalidomide and dexamethasone; CD = cyclophosphamide and dexamethasone; VCD = bortezomib, cyclophosphamide, and dexamethasone, NA = not available; ECG = electrocardiogram; U = undetectable; EED XR = esophagus, stomach, duodenum XR.

### Case 9

A 60-year-old man reported pain in his left hip in 2013, associated with weight loss. Multiple myeloma was diagnosed with multiple osteolytic lesions and a biopsy in the iliac lesion (Table 2). First-line treatment was CTD, with cyclophosphamide at 500 mg per week, thalidomide at 100 mg daily, and dexamethasone at 40 mg for eight cycles, and consolidation with ASCT was planned. Chemotherapy was started in December 2017, with eleven cycles performed until September 2018. Given the clinical response of myeloma, he was referred to evaluation by the transplant team when serology was positive for *T. cruzi* antigens. In October 2018, he was hospitalized due to disease progression, with a new bone lesion and significant abdominal distension. He was diagnosed with chagasic megacolon and his echocardiogram did not show abnormalities. His cPCR changed from negative in September to positive in November 2018, reaching 161.0 par Eq/mL in December 2018, when he dropped out. He evolved with several infectious complications, dying in January 2019.

### Review on Chagas disease reactivation and multiple myeloma

A total of eight cases of MM concomitant to Chagas disease were previously reported, as shown in Table 3. From these, four cases were monitored by parasitological methods and did not present positive parasitemia^18^. Another MM case presented two positive PCR for Chagas disease and received benznidazole^19^. Remarkably, only one case of CDR was confirmed in a MM patient 13 days after ASTC^20^. Other two cases were suspected of CDR^21,22^ during induction therapy before ASTC; the first was with a 100-fold increase of parasite copies by qPCR, without positive direct microscopy^21^. Another MM and Chagas disease patient with retinitis was suspected of CDR by two positive cPCR in the blood^22^. However, he survived without antiparasitic treatment and CDR was not confirmed based on standard recognized methods^22,24,25^.

**Table 3:** - *Trypanosoma cruzi* parasitemia in patients with multiple myeloma and Chagas disease with and without autologous stem cell transplants.

Article/country	Age - years, sex	Underlying disease	Clinical chronic form/Reactivation form	Immuno-suppressive drugs/related to CDR	Parasitemia during neutropenia Micro-Ht, blood culture	Serology pre/post-BNZ	Parasitemia post neutropenia or BNZ: Strout /blood culture	BNZ	Outcome
Altlcas^18^, AR	48 Male	Multiple myeloma	NA	NA	negative	+/- +390 d	negative/ negative	No	Survival 2 months
Altlcas^18^, AR	37 Female	Multiple myeloma	NA	NA	negative	+/discordant + 62d	negative/ negative	No	Dead 25 months
Altlcas^18^, AR	39 Male	Multiple myeloma	NA	NA	negative	+/+	negative/ NA	No	Survival 5 months
Altlcas^18^, AR	62 Male	Multiple myeloma	NA	NA	negative	+/+	negative/ NA	No	Survival 2 months
Pinazo^19^, BOL	44 Male	Multiple myeloma	Indeterminate	V, CA, C, P, Z	NA, PCR + 03/17/2010; 04/21/2020	+/NA	cPCR negative 05/04/2010	BNZ- 5 mg/kg/d – 60 days	Survival 12 months
Rojas^20^, CO	58 Male	ASCT Multiple myeloma	No symptoms/ Fever	8 cycles CR, L, D	Smear +13^th^ day ASCT	+/NA		BNZ - dose NA - 60 days	Survival 16 months transplant
Guiang^21^, ES	54 Female	Multiple myeloma ASCT	NA/no symptoms	CAR, E, M	qPCR - increased parasitemia/negative	+/+ (2 years later)	NA/NA qPCR negative	BNZ 5 mg/kg/d – 60 days	Survival 24 months
Conrady^22^, EC	69 Male	Multiple myeloma ASCT	Iridocyclitis: neutropenia	DA, L, D	Pan fungal PCR - 28 S rDNA – sequencing 97% identity *T. cruzi*	+/NA	Negative/ NA	No (patient denied)	Survival

Micro Ht = microhematocrit; BNZ = benznidazole; Parasitemia post = parasitemia post neutropenia or benznidazole treatment; AR = Argentina; NA = not available; BOL = Bolivia, cPCR = qualitative PCR; CO = Colombia; ASCT = autologous stem cell transplant; ES = El Salvador; EC = Ecuador; V = Vincristine; CAR = carmustine; M = melphalan; C = cyclophosphamide; P = prednisone; Z = zoledronate; CR = carfilzomib; L = lenalidomide; D = dexamethasone; E = Etoposide; DA = daratumumab.

Considering the degree of immunosuppression, the risk of CDR is proportionally higher in Allogeneic Stem Cell transplantation (44.4%) than in ASTC (8.3%), and in leukemia and lymphoma than in MM in ASCT and Allogeneic SCT settings^18^.

Table 4 shows three cases of CDR in patients undergoing ASCT. In patients with MM, one patient was described with CDR (Table 3)^20^. Two other cases of non-Hodgkin lymphoma had CDR 20 days before^18^ and 17 days post-transplant^33^. CDR was followed by survival in these three patients^18,20,33^, possibly related to the early diagnosis and treatment, and less aggressive immunosuppression. Table 4 shows the underlying disease associated with CDR under allogeneic SCT^18,34-36^. Most were diagnosed during graft-versus-host disease (GVHD) or chemotherapy; negative seroconversion for *T. cruzi* antibodies was noticed in four. All patients received antiparasitic treatment with benznidazole or nifurtimox (Table 4). In two single case reports, deaths were registered on the third and 30^th^ day of treatment^35,36^, the latter with chagasic panniculitis died due to the underlying disease. Despite the severity of immunosuppression in allogenic transplantation, it is noteworthy that all cases with prospective monitoring of parasitemia survived^18,34^.

**Table 4:** - Reactivation of Chagas disease associated with autologous or allogeneic stem cell transplantation

Article/ country	Age years, sex	Transplant/disease	Clinical form/serology	Reactivation	Diagnosis (+ day of transplant)	Negative seroconversion (before/after)	Immunosuppression (GVHD)	Antiparasitic Treatment	CDR: Parasitemia days post-therapy	Outcome
Rojas^20^, CO	58 Male	ASCT Multiple myeloma	No symptoms/ positive	Fever	Smear +13	positive/NA	8 cycles CF, L, D	BNZ - dose NA - 60 days	NA	Survival 16 months transplant
Altlcas^18^, AR	48 Male	ASCT (non-Hodgkin lymphoma)	NA/positive	yes	-20 + Strout neutropenia,	positive/positive	NA	BNZ - 5 mg/kg/d - 20 days	negative/ Strout	Survival 5 months
Challela^33^, CO	64 Male	ASTC/Non-Hodgkin Lymphoma	Indeterminate/positive	yes Fever	+ 17d Strout +131post therapy	positive/NA	3 cycles: E, ME, CYT, CIS, CAR	NFT- 120 mg/ day- 60 days BNZ -300 mg/d - 60 days	3 weeks negative Strout.	Survival soon after BNZ
Altclas^18^, AR	27 Male	Allogeneic SCT Chronic Myeloid Leukemia	Indeterminate/Positive	yes	+111 Strout	positive/negative	GVHD II +53 days	BNZ - 5 mg/kg/d at least 30 days	negative on 14^th^ day	Survival 71,3 months
Altclas^18^, AR	13 Male	Bone Marrow dysplasia	Indeterminate/positive	yes	+16 Strout	positive/negative	No	BNZ - 5 mg/kg/d at least 30 days	negative on 14^th^ day	Survival 33.3 months
Altclas^18^, AR	15 Male	Aplastic anemia	Indeterminate/positive	panniculitis	+178 Strout	positive/positive	GVHD II +92 days	BNZ - 5 mg/kg/d at least 30 days	negative on 14^th^ day	Survival 10 months
Altclas^18^, AR	27 Male	Bone Marrow dysplasia	Indeterminate/positive	yes	+120 Strout	positive/discordant	GVHD II+ 34 days	BNZ - 5 mg/kg/d at least 30 days	negative on 14^th^ day	Survival 13.3 months
Altclas^34^, AR	27 Male	Allogeneic SCT Chronic Myeloid Leukemia	Chronic/positive	Fever	+ 101 Strout	positive/negative IHA, ELISA, IFF cutoff	BU, C, CI, MT, CT	BNZ - 400 mg/d at least 35 days	Negative serology and parasitemia	Two years
Riganti^35^, AR	38 Male	Allogeneic SCT Chronic Myeloid Leukemia	No cardiac/positive	panniculitis	+69 Strout histopathology	NA	P - 60 mg/ TA - 5mg/12h	NFT– 600 mg/d – 30 days	7 days, fever, skin lesions	† 30 days hematological disease
Angheben^36^, IT	9 Female	Allogeneic SCT Acute lymphoblastic leukemia + Acute myeloid leukemia	No cardiac	Fever, hepatomegaly	+ 56 Smear	NA	C, ATG Prophylaxis CI, MT GVHD II P +26	BNZ - 10 mg/kg/d – 3 days	3 days	† 3 days

GVHD II = Graft vs Host disease; Negative seroconversion = positive serology changes to negative; BNZ = benznidazole; NFT = nifurtimox; CDR = Chagas disease reactivation; CO = Colombia; AR = Argentina; NA = not available; ASCT = autologous stem cell transplant; Allogeneic SCT = allogeneic stem cell transplantation; GVHD II = Graft vs. Host disease grade II; † = Death; CF = carfilzomib; L = lenalidomide; D = dexamethasone; E = etoposide; ME = methylprednisolone; CYT = cytarabine; CIS = cisplatin; CAR = carmustine; M = melphalan; B = Busulfam; C = cyclophosphamide; CI = cyclosporine; MT = methotrexate; CT = corticoid; ATG = anti-thymocyte globulin.

In addition, outside the transplant setting, CDR has been described as single case reports in 12 patients from 1968 to 2015 (Table 5), more frequently in non-Hodgkin lymphoma and Hodgkin lymphoma^37-48^. Reactivation occurred during the induction phase or chemotherapy in the majority. From these 12, seven received antiparasitic treatment with benznidazole or nifurtimox (Table 5), and only three survived after the end of the treatment^42,44,48^. The diagnosis was performed in three cases by necropsy^38,39,41^. Two patients died before 19 days of antiparasitic therapy, one by bacterial sepsis^46^ and the other after interruption of benznidazole due to leukopenia^45^. Therefore, this high lethality is possibly due to late diagnosis and significant impairment of the mechanisms involved in controlling the growth of parasites both by the underlying disease and chemotherapy.

**Table 5:** - Reactivation of Chagas disease in hematological diseases without transplantation.

Article/ Country/ transmission	Age-years, sex	Underline disease	Previous clinical form/serology	Reactivation	Diagnosis	Immunosuppression/ related to CDR	Antiparasitic treatment	CDR: Follow up (days)	Outcome (days)
Amato Neto^37^, BR. Vector, blood	35 Female	Acute myeloid leukemia	NA/+	Esophagus, pericardium, peritonium/+	Smear and fresh slide Trypomastigotes	NA	NA	8 days	†
França^38^, BR Vector	65 Male	Chronic Lymphoid	Chronic cardiac	Heart failure/Meningoencephalitis	Autopsy – Acute meningoencephalitis + chronic myocarditis	NA	No	No	†
Almeida^39^, BR Vector	26 Male	Hodgkin Lymphoma	Chronic cardiac//+	Reactivation (esophagitis)	amastigotes - lympho-mononuclear infiltrate/esophagus ↑↑ amastigotes	C, CT then C, MH	NA	No	†
Kohl^40^, ES Blood	14 Female	Lymphocytic leukemia	No cardiac/+	Reactivation	Smear, blood culture, xenodiagnosis, + serology	Reinstitution of chemotherapy	NFT- 15 mg/ kg/d 4 days (toxicity); NFT- 15mg/kg/d – 90 days	90 days Smear Negative/No symptoms or signals Polyneuropathy	Survival NA later
Metze^41^, BR Blood	46 Female	Hodgkin disease	Chronic cardiac/+	Acute myocarditis	Autopsy amastigotes	Induction NM, V, V, P, PRO	No	No	†
Simões^42^, Vector	42 Female	Non-Hodgkin disease	No cardiac	Acute myocarditis, esophagitis	Amastigotes biopsy esophagus	NA, soon after chemotherapy	BNZ – 300 mg/d -20 days	20 days, clinical improvement and normal histopathology	20 days interruption (leukopenia) Survival 6 months
Di Lorenzo^43^, AR, NA	9 Male	Acute lymphoblastic leukemia	NA/NA	Encephalitis	Amastigotes: brain parenchyma	CT	NFT doses and duration - NA	NA	†
Salgado^44^, BR Vector, blood	73 Male	Lymphocytic leukemia	No symptoms/+	Encephalitis	CSF - trypomastigotes	CH, CT	BNZ 300 mg/day – 60 days	NA	Survival 120 days
Rezende^45^, BR Vector	42 Female	Non-Hodgkin Lymphoma	Chronic cardiac, dysphagia	Acute myocarditis	Microscopy + peripheral blood. Amastigotes: larynx, digestive tract trachea,	C, H, V, P, BL – 6 months CDR - 30 days after the last cycle	BNZ - 10 mg/kg/d - 19 days	Clinical improvement Interruption due to leukopenia	Fever recurrence †
Oliveira^46^, BR Vector	67 Female	Non-Hodgkin Lymphoma	Chronic Cardiac and Digestive)/+	Reactivation Central Nervous System	CSF / blood	C, DO, V, P, MT, CYT - CDR 5 days after the last cycle	BNZ - 5 mg/kg/d † 8 days	Reduced parasitic load on the third day of therapy	† 8 days (bacterial sepsis)
Vicco^47^, AR, NA	65 Male	Non-Hodgkin Lymphoma	Chronic Cardiac/+	Reactivation Acute Chagas disease	Buffy coat +	D 16 mg/kg – CDR 10 days later	No	† 1 day	† 1 day
Garzon^48^, AR Vector	60 Male	Follicular lymphoma	Previous positive serology	Reactivation: high number of par Eq/mL	Blood smear Negative qPCR 577,959 par Eq/mL	5 cycles C, DO, V, P, R 7 days before the 6^th^ cycle	BNZ – 5 mg/kg/d 12/12h – 60 days	qPCR undetectable 1 month later.	Survival 60 days

CDR = Chagas Disease Reactivation; BR = Brazil; M = male; F = female; NA = not available; † = death; ES = El Salvador; BNZ = benznidazole; NFT = nifurtimox; AR = Argentina; CSF = cerebrospinal fluid; qPCR = quantitative PCR; C = Cyclophosphamide; CT = corticosteroids; MH = methyl-hydralazine; NM = nitrogen mustard; V = vincristine; P = prednisone; PRO = procarbazine; CH = Chlorambucil; H = hydroxydaunorubicin; BL = bleomycin; DO = doxorubicin; MT = methotrexate; CYT = cytarabine; D = Dexamethasone; DO = doxorubicin; V = vincristine; R = rituximab.

## DISCUSSION

In this review, the rate of Chagas disease reactivation registered in MM patients (1/8) is similar to that seen in ASCT^18^ (Tables 3 and 4) and lower than described in allogeneic stem cell transplants^18^ and hematological neoplasias (Table 5). Only one prospective study on MM and Chagas disease with four cases was published, in which parasitological methods monitored *T. cruzi* parasitemia^18^. Molecular methods monitored parasitemia in three case reports, two using cPCR^19,22^ and only one using qPCR^21^.

In our casuistic, no CDR was observed. In total, three out of the nine MM patients showed positive parasitemia; two had positive cPCR and qPCR, and none had CDR. This review suggests a low risk for CDR in these patients undergoing autologous ASTC, and our case series confirms that this is a rare event. CDR has been reported in only one MM + Chagas disease patient^20^ and two in non-Hodgkin lymphoma under ASCT^18,33^, as well as in seven patients with hematological disorders undergoing allogeneic hematopoietic stem cell transplantation^18,34-36^.

In addition, the frequency of positive parasitemia by parasitological or molecular methods in our casuistic is considerable and deserves careful management. Our findings indicate a 33.3% occurrence, which is higher than the 0% parasitemia observed in a case series of four MM patients using parasitological enrichment methods^18^. Notably, it is still lower than the 44.4% recorded in Allogeneic SCTH due to other hematological diseases^18^, as well as the 50–64% observed in *T. cruzi*/HIV coinfection^28,49,50^ and the 40–58.5% reported in chronic Chagas disease without known immunosuppression^28,51^.

A non-transplanted patient presented with 161.0 par Eq/mL and died due to complications of underlying disease before receiving benznidazole. In another patient, increased parasitemia was detected from the 28^th^ to the 33.5^th^-month post-ASCT, reaching 256 par Eq/mL. This level represents approximately a 50–80-fold increase compared to pre-transplantation values. Parasitemia decreased after antiparasitic therapy and was maintained at very low levels during 31 months of follow-up (Table 1). A similar case was reported in one MM patient 10 days after ASCT^21^. Finally, our third case had a positive blood culture and received benznidazole, followed by two negative cPCR during four months. This successful conversion of parasitological methods from positive to negative post-benznidazole was noticed before transplantation in a non-Hodgkin lymphoma patient without CDR^18,19^; however, the follow-up period was unknown.

The increased parasitemia could result from the dual action of MM therapy: the immunosuppression of Th1 and macrophage effector function, halting *T. cruzi* growth; and the increase of regulatory T cell function, allowing parasite growth^2,7-10^. On the other hand, drugs such as bortezomib are likely to help parasite killing by increasing natural killer cell function and stimulating INF-γ and TNF-α secretions^10^.

Remarkably, increased parasitemia was associated with the severity of the underlying disease and the response to chemotherapy in our cases. The three patients with parasitemia had advanced disease at diagnosis (DS IIIA or IIIB)^32^. One patient developed parasitemia during salvage therapy for relapsed MM, whereas another died soon after this detection, possibly due to MM progression, before receiving antiparasitic treatment. Moreover, most recently, the risk of MM at diagnosis has been defined by cytogenetic markers by hybridization *in situ* fluorescence (FISH), but this data was unavailable in our cohort. Additionally, in clinical practice, measuring the patient’s immunological status is unfeasible.

In addition, we reinforce the Argentinian clinical practice guidelines for Chagas disease^52^, which recommend parasitemia monitoring by qPCR in patients with Chagas disease associated with hematological malignancies.

Antiparasitic treatment has been recommended in the Brazilian Consensus on Chagas diseases^24^ for chronic Chagas disease patients. This treatment was successful for *T. cruzi–*HIV coinfected cases with increased parasitemia diagnosed by parasitological methods^27^. According to it, patients with MM and hematological diseases with increased parasitemia or positive parasitemia associated with higher immunosuppression must be considered for antiparasitic treatment with careful administration due to drug toxicity.

Regarding qPCR, the cut-off levels recommended for preemptive therapy are a matter of discussion. Our suggestion of ≥ 100 parEq/mL as the cut-off is based on patients with chronic Chagas disease and *T. cruzi*/HIV-infected patients with low parasitemia who did not present CDR^28,49^. Table 6 presents our suggestion of antiparasitic therapy for chronic Chagas disease + MM patients under or without ASCT, including shorter^53^ and intermittent regimens^54^. Although less toxic, their effectiveness is yet to be confirmed in chronic Chagas disease treatment, particularly in immunocompromised patients.

**Table 6:** - Antiparasitic treatment with benznidazole for chronic Chagas disease patients with multiple myeloma under or without autologous stem cell transplant.

Patient	Dose mg/kg/day	Period in days	alternatives	Comments
Chagas Disease Reactivation	adult 5-7 children 5-10	60	nifurtimox (if adverse events to benznidazole or resistance)	
≥ 100 par Eq/mL (qPCR), increasing parasitemia, or persistent parasitemia associated with severe immuno-suppression for rejection or transplant)	5- 7 at the most, 300 mg/day	30 to 60 days*	nifurtimox (if adverse events to Benznidazole or resistance)	
Conversion cPCR or qPCR from negative to positive	5-7 mg, at the most, 300 mg/day	30 days^53^	Intermittent regimen^54^	Torrico *et al.* ^53^ Álvarez *et al*.^54^

^53,54^These are low-toxicity schemes, whose effectiveness needs confirmation in chronic Chagas disease, particularly in immunocompromised patients; *in the absence of serious adverse events.

Finally, the strengths of this work include confirming the low CDR risk but finding a moderate risk of increased parasitemia in patients with MM and Chagas disease. Other strengths are the long follow-up period of parasitemia monitoring with molecular and parasitological methods in nine patients, comparison between parasitemia and clinical evolution of MM, and reduction of high parasitemia detected by qPCR after benznidazole therapy in one MM patient. As a limitation, this is not a controlled study comparing clinical and laboratory data. Moreover, the sample size is small but represents, to the best of our knowledge, the largest case series of MM and Chagas disease with parasitemia monitoring. Finally, the qPCR was conducted in-house with limitations such as a lack of automated processes at all stages and less comparability between laboratories.

## CONCLUSION

We found a considerable rate of increased parasitemia in MM patients under prospective monitoring by molecular and parasitological methods associated with severe progressive MM. No CDR was noticed, possibly due to antiparasitic treatment in light of higher parasitemia. We showed that molecular methods, including qPCR, should be recommended for *T. cruzi* parasitemia monitoring as markers for preemptive therapy. Moreover, we recommend the timely introduction of benznidazole to prevent CDR.
